# Estimating Costs and Benefits of CTL Escape Mutations in SIV/HIV Infection

**DOI:** 10.1371/journal.pcbi.0020024

**Published:** 2006-03-31

**Authors:** Vitaly V Ganusov, Rob J De Boer

**Affiliations:** 1 Theoretical Biology, Utrecht University, Utrecht, Netherlands; 2 Institute of Biophysics, Krasnoyarsk, Russia; Pennsylvania State University, United States of America

## Abstract

Mutations that allow SIV/HIV to avoid the cytotoxic T lymphocyte (CTL) response are well documented. Recently, there have been a few attempts at estimating the costs of CTL escape mutations in terms of the reduction in viral fitness and the killing rate at which the CTL response specific to one viral epitope clears virus-infected cells. Using a mathematical model we show that estimation of both parameters depends critically on the underlying changes in the replication rate of the virus and the changes in the killing rate over time (which in previous studies were assumed to be constant). We provide a theoretical basis for estimation of these parameters using in vivo data. In particular, we show that 1) by assuming unlimited virus growth one can obtain a minimal estimate of the fitness cost of the escape mutation, and 2) by assuming no virus growth during the escape, one can obtain a minimal estimate of the average killing rate. We also discuss the conditions under which better estimates of the average killing rate can be obtained.

## Synopsis

Due to their high mutation rate, RNA viruses—like SIV and HIV—can avoid recognition by the host immune response by evolving new variants (i.e., immune escape mutants). Avoiding the cytotoxic T lymphocyte (CTL) immune responses is one of the major obstacles for the development of vaccines to HIV, and this avoidance seems a major mechanism of HIV disease progression to AIDS. Using a relatively general mathematical model, Ganusov and De Boer suggest a simple technique by which two main parameters determining the likelihood of viral escape can be estimated. First is the “cost” of the escape mutation, which is the relative fitness reduction in the virus replication rate. Second is the rate at which the CTL response specific for one epitope “clears” virus-infected cells. Application of their technique to data on virus escape helps to quantify the costs and benefits of CTL escape mutations in SIV/HIV infection.

## Introduction

Several observations suggest that cytotoxic T lymphocyte (CTL) responses play an important role in controlling virus replication in SIV/HIV infections. First, depletion of CD8^+^ T cells during chronic SIV infection of rhesus macaques leads to a rapid increase in viral loads [1], and depletion of CD8^+^ T cells prior to SIV infection results in rapid progression and death of animals following infection [2]. Second, the rate of progression of HIV-infected individuals is strongly dependent on MHC heterozygosity and specific MHC class I alleles [3–6]. Finally, HIV infection of humans and SIV infection of monkeys often results in evolution of viral mutants that are not recognized by the specific CTL responses [7,8]. Many such mutants, although not all, result from point mutations in epitopes presented by the host MHC class I molecules and recognized by the CTL response [7,9,10]. While this evidence suggests an important role of CTL responses in controlling virus replication, studies quantifying the selection pressure imposed by the CTL response on the virus population, as well as the costs suffered by mutants evading the CTL response, have just recently become available. Two recent studies employ a simple way of estimating these two parameters [11,12]. The fitness cost of a CTL escape mutant is generally investigated in “reversion” experiments by observing the dynamics of the mutant in hosts lacking the MHC class I allele presenting the wild-type, unmutated epitope. The average rate, *R,* at which the logarithm of the ratio of the wild-type to the mutant frequency increases with time, is interpreted as the cost of the escape mutation [8, 12,13]. Previously it was shown that this rate provides an estimate of the absolute difference between replication rates of the wild-type virus and the mutant, and not of the relative difference (i.e., relative fitness). The estimated absolute rate difference strongly depends on the viral replication rate [14], and this makes it difficult to compare how “costly” the different CTL escape mutations are.

During “escape” experiments in which a wild-type virus is substituted with a mutant, the average rate, *E,* at which the logarithm of the ratio of the wild-type frequency to the mutant decreases with time, is calculated. The sum of the two rates, *R* + *E,* provides an estimate of the CTL killing rate, or the rate at which cells productively infected with the wild-type virus are killed by the CTL response due to the expression of the non-mutated epitope [11,12]. In the derivation of these results, the authors made an implicit assumption that the virus replicates at a constant rate, and that the rate at which the CTL response clears virus-infected cells is constant, or declines slowly over time. Given recent findings on the viral dynamics during the acute phase of SIV/HIV infection [7,15,16], both assumptions are likely to be simplifications. In this paper we show that if these assumptions are violated, estimation of the cost of escape mutations and killing rates is more complex. Using a relatively general model for the dynamics of the wild-type and mutant viruses in a given host (see Materials and Methods), we provide a theoretical basis for obtaining minimal estimates for these parameters using in vivo data.

## Results

### Reversion Experiments

In the reversion experiments, the dynamics of the CTL escape mutant is observed in a host lacking the MHC class I allele presenting the wild-type epitope. In such a host, the CTL response specific for the wild-type epitope is absent, i.e., *k*(*t*) = 0 in Equation 8. Since the ratio of the wild-type virus to the mutant should change exponentially (see Equation 8), it is useful to calculate the average rate *R* at which the logarithm of the ratio *z*(*t*) increases with time. If the measurements of the ratio *z*(*t*) are available at two time points, *t_s_* (start) and *t_e_* (end), with corresponding measured ratios *z_s_* = *z*(*t_s_*) and *z_e_* = *z*(*t_e_*), the average replacement rate *R* of the mutant by the wild-type in the time interval (*t_s_, t_e_*) can be calculated from the data and the model as follows


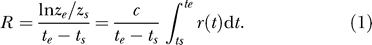


**Figure 1:**
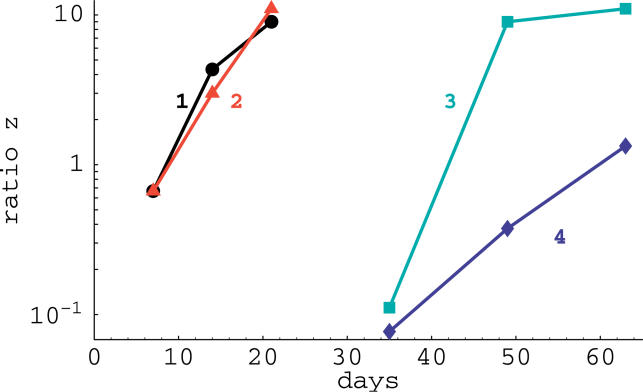
Reversion of the Gag_206−216_ SIV Mutant to the Wild-Type Virus in Cynomolgus Macaques during the Acute Phase of SIV Infection Animals 1 and 2 were infected with both the mutant and wild-type virus at the same time, animals 3 and 4 were infected only with the mutant, and it took about a month before the wild-type virus appeared. Using Equation 1, we estimated the average rate of replacement *R* = 0.19, 0.20, 0.16, 0.10 d^−1^ for these four cases, respectively (*t_s_* = 7 d, and *t_e_* = 21 d for animals 1 and 2, and *t_s_* = 35 d and *t_e_* = 63 d for animals 3 and 4). Replacement occurred at a higher rate early during the acute infection, which is readily explained by a faster rate of virus replication at these earlier time points. Assuming exponential growth of the virus at days 7 through 21 with *r_max_* = 1.5 d^−1^ using Equation 2, we obtain the minimal estimate of the cost of the Gag_206−215_ escape mutation *c ≈* 13%. If there were no changes in the cost *c* in animals 3 and 4 after 63 d during the experiment, these data would suggest a reduction in the average replication rate of the virus between days 7–21 and 35–63 of approximately [(0.20 + 0.19) *=* (0.16 + 0.1)] = 1.5-fold, which is not unrealistic. Note that the frequency of the CTL escape mutant in the virus population at time *t* is given by 1*=* (1 + *z*(*t*)).

Note that to estimate the rate *R,* or other parameters below, from the data, one needs to have at least two time points in which both viral variants are present—when more than two time points are available and the changes in the death rate *d*(*t*) with time are known, more sophisticated techniques could be used for parameter estimation [17]. Importantly, Equation 1 implies that to estimate the cost of the escape mutation *c* one needs to know how the virus replication rate, *r*(*t*), changes over the time of the experiment. Unfortunately, this replication rate is generally unknown. By comparing the rates of replacement of various escape mutants with the wild-type in different reversion experiments, it is often concluded that the mutant with the highest reversion rate *R* has the highest cost [7,8,12]. Our analysis suggests that differences in the rates of replacement may also arise due to differences in the virus replication rate during the experiment. For example, acute SIV/HIV infection is characterized by three different phases: initial expansion, contraction, and a relatively stable level of the virus population. It seems likely that the replication rate of the virus differs in these three phases. The virus is likely to replicate at the highest rate early during infection (*r*(*t*) *≈* 1.5 d^−1^), the rate of replication may be lower around the peak of viremia (*r*(*t*) *≈* 1 d^−1^), and is likely to approach its lowest value during the stable phase (*r*(*t*) *≈* 0.5 d^−1^) [18–21]. Reversions that take place during these three different phases may therefore have quite different replacement rates. In particular, we expect the rate of reversion during the first phase to be higher than that during the third phase. This prediction is consistent with recent data of Kobayashi et al. [22] in which the replacement of a CTL escape mutant by the wild-type occurred at a faster rate between days 7 and 21 than between days 35 and 63 post-infection, shown in Figure 1.

Nevertheless, even if the changes in virus replication rate over time are not known, one can make a minimal estimate of the fitness cost of the escape mutation. By assuming that during the experiment the virus population expands exponentially at a fixed maximal rate *r*(*t*) = *r_max_,* the minimal estimate of fitness cost is found using Equation 1


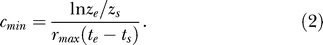


**Figure 2:**
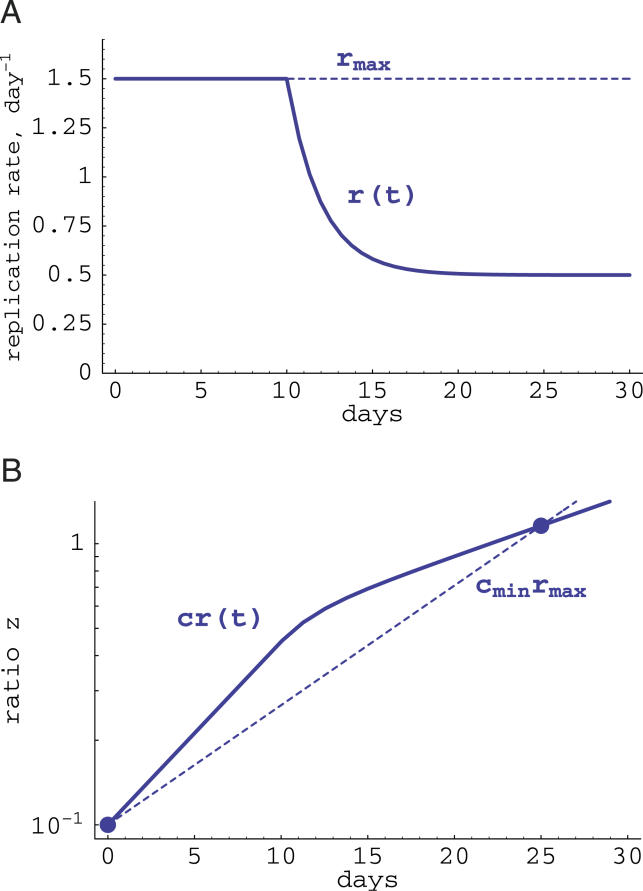
Estimating the Cost of a CTL Escape Mutation (A) Plots the hypothetical changes in the replication rate of the virus during the acute phase of infection. We assume that initially (until time *T_r_*) the virus population expands exponentially at rate *r_max_*, and after *t* = *T_r_ ,* the virus replication rate is reduced exponentially at rate λ to a smaller value *r_min_*. Biologically this would correspond to the acute phase of SIV/HIV infection when the replication rate of the virus depends on the availability of target cells, which are depleted by the virus. (B) Plots changes in the ratio *z*(*t*) calculated using Equation 8 with *k*(*t*) = 0 for the replication rate *r*(*t*) shown by a solid line in (A). For two measurements of the ratio *z* at *t_s_* = 0 and *t_e_* = 25 d, we obtain a minimal estimate of the cost of the escape mutation assuming exponential virus growth with *r_max_* = 1.5 d^−1^ (shown by dashed lines), i.e., *c_min_* = 0.065 which is 65% of the actual fitness cost. Other parameters: *r_min_* = 0.5 d^−1^, *T_r_* = 10 d, λ = 0.5 d^−1^, *z*(0) = 0.1, *c* = 0.1.

The fact that Equation 2 provides an underestimate of the fitness cost is demonstrated in Figure 2. In this example, the cost of the escape mutation is fixed at 0.1 (10% relative reduction in the replication rate of the mutant). We assume that during the infection, the replication rate is maximal before the peak of the infection, and then is reduced to a smaller value (e.g., due to decreased target cell availability). Due to this reduction in the replication rate, the replacement of the mutant by the wild-type virus also becomes slower after the peak of viremia (Figure 2). However, if the measurement of the mutant and wild-type frequencies were done before and after the peak of viremia, one would underestimate the cost of the mutation when assuming a fixed maximal replication rate of the virus (in Figure 2 the estimated *c_min_* ≈ 0.065).

Two additional points needs to be stressed. It is often concluded that the time taken for replacement of the mutant by the wild-type in reversion experiments is related to the cost of the escape mutations, i.e., longer reversion times from the start of experiment to a complete reversion correspond to lower fitness costs (e.g., [23]). Clearly, when only mutant virus infects a new host, it will take some time before the wild-type is generated by reverse mutation. Our analysis demonstrates that the *rate* of replacement of the mutant by the wild-type, and not the *time* to a complete reversion, is proportional to the fitness cost of the escape mutation. Therefore, as an approximate measure of the fitness cost of an escape mutant one should only consider the time period during which the actual substitution of the mutant by the wild-type took place. In many situations, this is the period between the first time the wild-type replaces the mutant (i.e., 100% wild-type sequences are observed) and the last time when only the mutant is present (i.e., 0% wild-type sequence is observed).

We have shown that the rate of replacement is determined by the fitness cost *c* and the virus replication rate *r*(*t*), and that changes in the rate of replacement are likely to occur due to changes in the replication rate. Several studies have documented that the fitness cost suffered by CTL escape mutants can be reduced by additional compensatory mutations [24–26]. Changes in the fitness cost are likely to affect the rate of replacement of the mutant by the wild-type during reversion experiments. Since in acute SIV/HIV infections, these replacements appear to occur faster than in chronic infections [12], accumulation of compensatory mutations during chronic infections is more likely to occur. This in turn could lead to a reduction in the fitness cost, slower replacement kinetics, and consequently, to underestimation of the initial fitness cost.

### Escape Experiments

During escape experiments, the wild-type virus is subjected to additional killing rate *k*(*t*), and the mutant virus suffers a fitness cost *c*. To characterize the dynamics of substitution of the wild-type by the mutant, it is useful to estimate the “escape” rate *E* at which the logarithm of the ratio of the wild-type frequency to the mutant decreases with time. This rate can be calculated from the data and in the model given in Equation 8


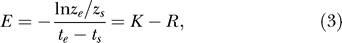


where 
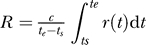
 is the average difference in replication rates of the wild-type and the mutant, and 
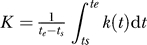
 is the average killing rate over the time period (*t_s_, t_e_*) during the escape; *z_s_* and *z_e_* are the measured ratios of the density of the wild-type to the mutant virus available during the escape experiment at two time points *t_s_* and *t_e_,* respectively. Equation 3 has a simple biological interpretation: the rate of replacement of the wild-type by the mutant in escape experiments is given by the difference between the average killing rate (at which the wild-type virus is cleared by the CTL response) and the average difference in the replication rate of the wild-type virus and the mutant (at which the wild-type overgrows the mutant).

Two groups have independently proposed to use escape experiments to estimate the rate at which the CTL response kills cells expressing the wild-type CTL epitope [11,12]. The average killing rate *K* can be calculated from Equation 3


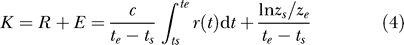


Importantly, Equation 4 suggests that in order to determine the average killing rate one not only needs to have an estimate of the cost of the escape mutation *c*, but also to know changes in the replication rate of the virus *r*(*t*) with time during the escape experiment. The latter, again, is generally not known. In the absence of such knowledge, two estimates of the average killing rate are possible. First, one could assume that during the escape experiments, there is no virus growth. Letting *r*(*t*) = *R* = 0, one finds a minimal estimate of the average killing rate *K_min_,* which is equal to the rate of substitution of the wild-type virus by the mutant in escape experiments, i.e., one obtains *K_min_* = *E*.

**Figure 3:**
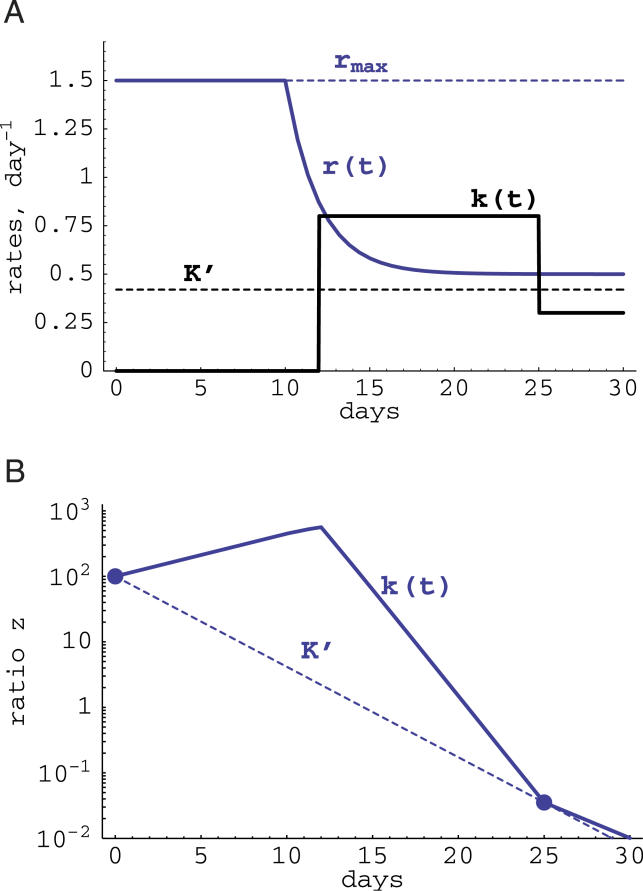
Estimating the Average Killing Rate (i.e., the Rate at Which a CTL Response Specific to One Viral Epitope Clears Virus-Infected Cells) (A) Plots an example of the replication rate *r*(*t*) and the killing rate *k*(*t*) occurring during the acute phase of an infection. The replication rate is identical to that in Figure 2. (B) Plots the changes in the ratio of the wild-type virus to the mutant density as a function of time. Thick solid lines denote the case where the replication rate *r*(*t*) and the killing rate *k*(*t*) change with time in accord with continuous lines in (A). For two measurements of the ratio *z* at *t_s_* = 0 and *t_e_* = 25 d, we estimate the average killing rate using Equation 5, assuming constant rate of virus replication with *r*(*t*) = *r_max_* during both reversion and escape experiments, shown by thin dashed lines in (A) and (B) and in Figure 2A, estimated *K′* = 0.42 d^−1^. Note that this estimate of the average killing rate underestimates the maximum killing rate *k_max_*. To describe the immune response we let *T_on_* = 12 d, *T_of_ =* 25 d, *k_max_* = 0.8 d^−1^, *k_min_* = 0.3 d^−1^. Other parameters are the same as in Figure 2 and *z*(0) = 10^2^.


Second, one could assume that during both the reversion and escape experiment, the virus replication rate is constant and the same (i.e., *r*(*t*) = *r*), and that the CTL response clears virus-infected cells at a constant rate *K′* (Figure 3). For these assumptions, the difference in replication rates of the wild-type and the mutant viruses is the same during escape and reversion experiments, *(R = cr),* and one can combine Equations 1 and 4 to find that the average killing rate *K′* is the sum of the rate of replacement of the mutant by the wild-type virus in reversion experiments (given by Equation 1), and the rate of replacement of the wild-type virus by the mutant in escape experiments, i.e.,

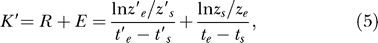


where *z′_s_* and *z′_e_* are the ratios of the wild-type virus to the mutant density at times *t′_s_* and *t′_e_* during the reversion experiment. This actually represents the calculation performed in [11]. While using this result, however, one should remain careful and realize that adding parameters *R* and *E* estimated in different hosts may be an oversimplification since even genetically similar individuals may differ in their responsiveness to the same pathogen, in part due to different T cell receptor repertoires [27].

While Equation 3 delivers the minimal estimate of the average CTL killing rate, the rate *K′* given in Equation 5 may overestimate or underestimate the actual average killing rate *K* (demonstrated in Table 1). This is due to the fact that the rate of virus replication may be different in reversion and escape experiments. If during the reversion experiments, the viruses replicate at a higher rate than during the escape experiments, Equation 5 will overestimate the average killing rate (Table 1, second row). This is likely to happen if the reversion occurs before the peak of viremia (when the virus replication rate is likely to be maximal), and the escape occurs after the peak of viremia (when the virus replication rate is likely to be reduced). In several published studies on CTL escape and reversion during acute SIV infection of macaques this is indeed the case [11,22,28]. By obtaining estimates *K_min_* and *K′* one can thus find the minimal and maximal estimate of the average killing rate, respectively (Table 1, second row). In a recently published study on SHIV escape in pigtail macaques [11], the authors found that during the escape (which occurred after the peak of viremia) the conservative estimate of rate of substitution of the wild-type virus by the CTL escape mutant KP9 (SIV Gag_164−172_) was *E ≈* 0.71 d^−1^. During the reversion (which occurred before the peak of viremia), the mutant was substituted by the wild-type at a rate *R ≈* 0.38 d^−1^. By assuming that the rate of virus replication is higher before the peak of viremia than that after the peak, we obtain the following minimal and maximal estimates of the average killing rate of the CTL response specific for the KP9 epitope (*K_min_, K′*) = (0.71 d^−1^, 1.09 d^−1^).

**Table 1:**
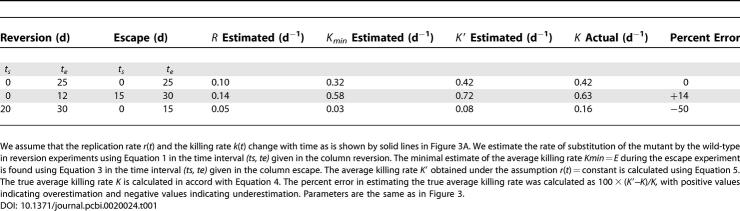
Estimating the Average Killing Rate

If during the reversion experiments the rate of virus replication is lower than that during the escape experiments (Table 1, third row), Equation 5 will underestimate the average killing rate. This is likely to happen if the reversion occurs much later than the escape. Finally, by assuming that the CTL killing rate is constant over time and estimating *K_min_* and/or *K′,* one always underestimates the maximal killing rate if the killing rate *k*(*t*) changes over time (compare estimates obtained in Table 1 and *k_max_* given in the legend to Figure 3).

## Discussion

In this paper we have provided a theoretical basis for estimating the costs of CTL escape mutations and the average rate at which the CTL response specific for a given epitope clears virus-infected cells. We show that by assuming exponential growth of the virus during the reversion experiments, one can obtain a minimal estimate for the cost of escape mutation (using Equation 2). Similarly, by assuming no virus growth during the escape experiments, one can obtain the minimal estimate of the average rate at which the CTL response specific to one viral epitope clears virus-infected cells (using Equation 3). Since our model is relatively general, our conclusions about estimating the costs and benefits of CTL escape mutations of SIV/HIV are equally applied to acute and chronic phases of SIV/HIV infection. However, while during the acute phase there are likely to be substantial changes in the rate of virus replication *r*(*t*) and the killing rate *k*(*t*) [7,15,16], we expect much smaller changes in these parameters with time in the chronic phase. This in turn implies that estimates of the cost of the escape mutation and the average CTL killing rate obtained during the chronic phase of SIV/HIV infection may be less affected by changes in the rate of virus replication. To understand the validity of the estimates obtained we need to better understand the dynamics of the virus and the effective CTL response during the chronic phase of SIV/HIV infection in a given host. A better understanding of how escape mutations affect the fitness of mutants (by reducing the replication rate and/or by increasing the death rate of virus-infected cells), and the mechanisms by which the CTL response controls the virus replication (lytic and/or nonlytic) will help in obtaining better estimates of the costs and benefits of escape mutations in SIV/HIV infection.

## Materials and Methods

### Main model.

We assume the following scenario for viral escape. The wild-type virus has a higher replication rate, and cells infected with the wild-type virus are killed at a higher rate by the CTL response. The mutant virus has a lower replication rate and cells infected with the mutant virus are killed at a lower rate by the CTL response. We formulate a mathematical model describing the dynamics of the density of cells productively infected with the wild-type *w*(*t*) or the mutant *m*(*t*) viruses, when both viral variants are present in an infected host. The model is given by the following equations


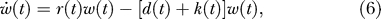



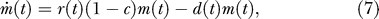


where *r*(*t*) and *r*(*t*)(1−*c*) is the replication rate of the wild-type and the mutant, respectively*, c* is the cost of the escape mutation defined as a selection coefficient, *d*(*t*) is the per capita clearance rate of both variants (which is typically equal to the death rate of productively infected cells, see below), and *k*(*t*) is an extra death rate at which virus-infected cells expressing the wild-type CTL epitope are being cleared by the epitope-specific CTL response. Since both SIV and HIV particles are known to be short-lived in vivo [29–31], densities of virus particles are likely to be proportional to the densities of cells productively infected with each virus variant given in Equations 6 and 7. Note that in this model we ignored additional mutations, and focus on the replacement kinetics. We also analyze two additional models on virus escape (see below). In one model, we let the escape mutant be less fit due to an increased clearance rate of the cells infected with the mutant virus. In another model, the CTL response controls virus growth nonlytically, by reducing the rate of virus replication.

It is useful to rewrite Equations 6 and 7 to describe the dynamics of the ratio of the wild-type to mutant density *z*(*t*) *= w*(*t*) */ m(t*):


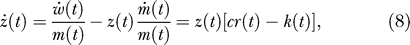


where *cr*(*t*) is the absolute difference in the replication rates of the wild-type and the mutant [13,14]. Thus we find that the ratio *z*(*t*) of density of the wild-type virus to the mutant changes exponentially with the time-dependent per capita rate *cr*(*t*) *− k*(*t*) determined by fitness cost of the escape mutation *c* times the rate of replication of the wild-type *r*(*t*), and the magnitude of the immune response directed against the wild-type epitope *k*(*t*). Importantly, CTL responses to other epitopes of both wild-type and mutant viruses cancel out in Equation 8, and the dynamics of the ratio *z*(*t*) is dependent only on the CTL response to the wild-type epitope. This could be different in other models, for example, where CD8^+^ T cell responses reduce the rate of virus replication by noncytolytic mechanisms (see below). In the main text we consider how the cost *c* and the killing rate *k*(*t*) can be estimated using in vivo data.

### Cost of mutation results in higher death rate of the mutant.

We consider the case when the escape mutation renders the mutant less fit due to an increased death rate of virus-infected cells. The dynamics of cells productively infected with the wild-type *w*(*t*) and the mutant *m*(*t*) viruses is given by the following equations:


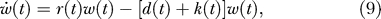



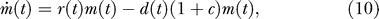


where *c* is the cost of the escape mutation. Rewriting Equations 9 and 10 for the ratio of the wild-type to mutant *z*(*t*) *= w*(*t*) */ m(t*) for times when both virus variants are present in the host, we obtain:


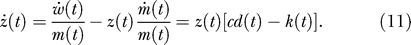


To estimate the cost of the escape mutation and the average CTL killing rate, one needs to know the changes in the death rate of the cells productively infected with the virus with time. Since these changes will be dependent on the CTL responses specific to both wild-type and mutant viruses, the death rate *d*(*t*) is likely to change during the acute phase of SIV/HIV infection, and may be relatively constant in the chronic phase.

### Noncytolytic CD8^+^ T cell response.

The model describing the dynamics of the cells infected with the wild-type *w*(*t*) and the mutant *m*(*t*) viruses, is given by the following equations:


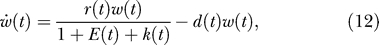



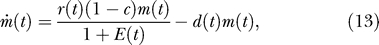


where the assumptions on the virus growth are similar to those in the main model. However, we assume that the CD8^+^ T cell response reduces the rate of virus replication. The reduction in the replication rate is due to the CD8^+^ T cell response specific for the wild-type epitope *k*(*t*) and due to CD8^+^ T cell responses to other viral epitopes *E*(*t*)*.* Note that in contrast with the main model, the parameter *k*(*t*) is now dimensionless. As in the main text, we write an equation for the dynamics of the ratio of the density of the wild-type to the mutant *z*(*t*):


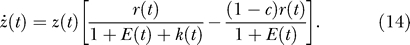


During the reversion experiments, the specific CD8^+^ T cell response directed against the wild-type epitope is absent (i.e., *k*(*t*) = 0), and the change in the ratio *z*(*t*) is given by


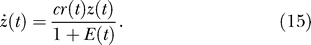


Assuming that the virus replicates at the maximum rate *rmax* in the absence of the CD8^+^ T cell response (*E*(*t*) = 0), one recovers the same equation for *z*(*t*) as is given in Equation 8 at *k*(*t*) = 0. This suggests that Equation 2 also gives the minimal estimate of the fitness cost of an escape mutant.

During the escape experiments Equation 14 holds. One can rewrite this equation:


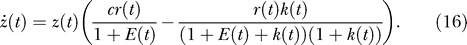


While this expression is somewhat complex, its interpretation is similar to that of Equation 8: the first term on the right hand side corresponds to increase in the frequency of the wild-type due to cost of escape mutation, and the second term corresponds to a decrease in the frequency of the wild-type due to the CD8^+^ T cell response specific for the wild-type epitope.

There are two differences with the equation found in the main model, however. First, we find that the rate of accumulation of the mutant in the population depends on the replication rate of the virus: a higher replication rate *r*(*t*) corresponds to a faster accumulation. Therefore, slow accumulation of the escape mutants in some experiments may reflect slow replication of the virus. This is in contrast to the prediction found when the CD8^+^ T cell response controls the virus by killing virus-infected cells, where the opposite trend is observed (i.e., the faster rates of virus replication leads to slower replacement of the wild-type by the mutant virus, see Equation 3).

Second, there is no an easy way of estimating the CD8^+^ T cell suppression efficacy *k*(*t*) unless changes in the total response *E*(*t*) and replication rate *r*(*t*) with time are known. Only by assuming that the growth rate and the two immune responses are constant over time, can such an estimate be made. As discussed above, this estimate, however, will also depend on how well the assumptions of the constancy of the immune response and of the growth rate over time are satisfied.

## Abbreviations

*c*: fitness cost
CTL: cytotoxic T lymphocyte
*E*(*t*): total response
K: average killing rate
*k*(*t*): killing rate
*m*(*t*): mutant
*r*(*t*): virus replication rate
*w*(*t*): wild-type
